# The Impact of Diesel Oil Pollution on the Hydrophobicity and CO_2_ Efflux of Forest Soils

**DOI:** 10.1007/s11270-018-3720-6

**Published:** 2018-02-04

**Authors:** Edyta Hewelke, Jan Szatyłowicz, Piotr Hewelke, Tomasz Gnatowski, Rufat Aghalarov

**Affiliations:** 1 Laboratory - Water Centre, Faculty of Civil and Environmental Engineering, Warsaw University of Life Sciences – SGGW, Ciszewskiego 6, 02-776 Warsaw, Poland; 2 Department of Environmental Improvement, Faculty of Civil and Environmental Engineering, Warsaw University of Life Sciences – SGGW, Nowoursynowska159, 02-776 Warsaw, Poland

**Keywords:** Physical properties of soil, Diesel contamination, Soil water repellency, Soil moisture content, CO_2_ efflux

## Abstract

The contamination of soil with petroleum products is a major environmental problem. Petroleum products are common soil contaminants as a result of human activities, and they are causing substantial changes in the biological (particularly microbiological) processes, chemical composition, structure and physical properties of soil. The main objective of this study was to assess the impact of soil moisture on CO_2_ efflux from diesel-contaminated albic podzol soils. Two contamination treatments (3000 and 9000 mg of diesel oil per kg of soil) were prepared for four horizons from two forest study sites with different initial levels of soil water repellency. CO_2_ emissions were measured using a portable infrared gas analyser (LCpro+, ADC BioScientific, UK) while the soil samples were drying under laboratory conditions (from saturation to air-dry). The assessment of soil water repellency was performed using the water drop penetration time test. An analysis of variance (ANVOA) was conducted for the CO_2_ efflux data. The obtained results show that CO_2_ efflux from diesel-contaminated soils is higher than efflux from uncontaminated soils. The initially water-repellent soils were found to have a bigger CO_2_ efflux. The non-linear relationship between soil moisture content and CO_2_ efflux only existed for the upper soil horizons, while for deeper soil horizons, the efflux is practically independent of soil moisture content. The contamination of soil by diesel leads to increased soil water repellency.

## Introduction

Soil pollution with petroleum hydrocarbons is a major environmental problem. Soil may become contaminated with these hydrocarbons by various routes, such as leakage from underground storage tanks and pipelines, accidental spills during transportation, drilling sites and improper waste disposal practices. According to Panagos et al. (2013), the number of estimated potential contaminated sites in the European Union is more than 2.5 million, with around 342 thousand identified contaminated sites. Mineral oil and heavy metals are the main contaminants, contributing approx. 60% to soil contamination. In Poland, an average of 2.5 thousand accidents related to the leakage of petroleum substances are recorded each year (Rakowska et al. 2012). The presence of petroleum hydrocarbons in soils is a problem that has caused concern worldwide because it poses a huge threat to human health and natural ecosystems.

Diesel oil is a complex petroleum hydrocarbon which is obtained during crude oil distillation, and is made up of low molecular weight alkanes and polycyclic aromatic hydrocarbons (Kaur et al. 2015). The hydrocarbon composition of diesel fuel makes it toxic to the environment and its widespread application in human activity makes diesel fuel one of the most hazardous hydrocarbon pollutants (Muratova et al. 2012).

The contamination of soils by hydrocarbons disturb biological and particularly microbiological, chemical and physical properties of soils, including their wettability (Klamerus-Iwan et al. 2015; Rodríguez-Rodríguez et al. 2016). The hydrophobic nature of hydrocarbons can modify the wettability of the surface of soil particles and they thus contribute to soil water repellency when coating soil particles (Roy et al. 1999). Soil water repellency affects hydrological and ecological soil functions by decreasing water infiltration, increasing surface runoff and erosion, and impeding plant growth (Doerr et al. 2000). Water repellency of soils limits their water sorptivity and results in uneven moisture distribution, forming preferential water flow in the soil profile (Dekker and Ritsema 1996; Szatyłowicz et al. 2007; Hewelke et al. 2016). The phenomenon of soil hydrophobicity has long been known and intensively studied in naturally occurring soils (DeBano 2000; Doerr et al. 2000). Oil contamination strongly increases the hydrophobicity of the soil; it loses its ability to absorb and retain water, displacing the air from the soil pores and ultimately destroying the water and air regime, leading to enhanced surface runoff, erosion and reduced soil moisture (Adams et al. 2008; Marín-García et al. 2016). Aislabie et al. (2004) noted that hydrocarbon-contaminated soils were weakly hydrophobic in the Antarctic region, but impacts on moisture retention were negligible. Flowline additives associated with oilfield installation on Barrow Island, Australia, in the observations of George et al. (2011), were shown to have had no effect on water repellency. Most studies dealing with hydrocarbon-induced soil hydrophobicity focused mainly on crude oil and oil pollution (Roy and McGill 2000; Adams et al. 2008; Lourenço et al. 2015; Marín-García et al. 2016; Gordon et al. 2018) and, to the best of our knowledge, only Takawira et al.’s (2014) study was conducted for diesel polluted soils. In the findings of the latter study, hydrocarbon contamination induces water repellency and reduces moisture retention on inherently wettable tropical sandy soils. The water from storms falling on initially dry and recently contaminated soils may trigger contaminant transport and erosion via enhanced surface runoff, and lead to the rapid spreading of contaminants once they reach the groundwater systems, confirming that hydrological impacts are critical for the recovery of contaminated sites. Sawatsky and Li (1997) suggested that water repellency should be included in the assessment of hydrocarbon-contaminated soils. This is because the failure of bioremediation may be attributed to the physical properties of soil negatively influenced by hydrocarbon residuals, and especially to the ability of soil to absorb water due to water repellency (Li et al. 1997). In summary, the findings of these earlier studies about soil water repellency in hydrocarbon-contaminated soil are inconsistent and often contradictory.

Soil contamination with hydrocarbons increases total carbon content in soil. When hydrocarbons are mineralized by microorganisms, CO_2_ is the main product. The native microbial population of soils can adjust their metabolism in order to use organic contaminants as carbon and energy sources (Szarlip et al. 2014). Measuring CO_2_ production or O_2_ consumption can provide an accurate measure of the biodegradation kinetics of contaminants in soils (Baptista et al. 2005; Kim et al. 2005; Muratova et al. 2012). These parameters, especially CO_2_ production, are used extensively to estimate the process of remediation of contaminated soils. Several studies linked CO_2_ production with the rate of degradation of contaminants under laboratory conditions (Sharabi and Bartha, 1993; Schoefs et al. 2004; Baptista et al. 2005; Van De Steene and Verplancke 2007) and at contaminated field sites (Sihota et al. 2010, 2016; Noel et al. 2016).

Soil moisture content is one of the most important environmental factors driving productivity and carbon cycling in terrestrial ecosystems. Next to temperature, it is a primary determinant of the rate at which soil carbon is mineralized by microbes into CO_2_. The relationship between soil moisture and soil respiration is known to be variable in naturally occurring soils, and has been described by numerous non-linear functions, where soil water is expressed as gravimetric, volumetric moisture content, fractions of water holding capacity and water-filled pore space (Moyano et al. 2012, 2013). Relatively few studies have dealt with the effect of moisture contents on soil CO_2_ efflux from diesel-polluted soils (Ferguson et al. 2003; Horel and Schiewer 2009). The mentioned studies deal with soils from cold regions (Antarctic and Alaskan soils) and show that the soil moisture content has a minor effect on CO_2_ efflux.

On the other hand, a study by Goebel et al. (2005), in which carbon mineralization from different (not contaminated) topsoil horizons was related to soil wettability, revealed that CO_2_ efflux rates decreased as soil water repellency increased. Lamparter et al. (2009), however, found that CO_2_ release rates decreased as soil water repellency increased, whereas no significant relationship could be observed for the same soils coming from a moist state and dried to − 31.6 kPa.

Soils are complex systems with long-lasting resilience and system reaction, but with limited regeneration capacities after mismanagement, especially contamination. An understanding of the hydrophobic compounds in the soil matrix and their interaction with soil moisture under the effects of hydrocarbon contamination of soil are needed in order to better plan and carry out remediation processes.

The aim of the study was to determine the effect of diesel oil contamination on soil wettability and CO_2_ efflux from selected forest soils under a temperate climate. The study involved the evaluation of the influence of different amounts of diesel oil contaminations on soil water repellency and CO_2_ efflux with the effect of soil moisture changes. We hypothesized that (1) the contamination of soil by diesel oil leads to increased soil water repellency, (2) the CO_2_ efflux response differs for natural and hydrocarbon-contaminated soils and (3) the CO_2_ efflux is related to soil moisture content changes.

## Materials and Methods

### Study Site and Basic Soil Properties

This study was conducted on soil from the Chojnów Forests in east-central Poland, which is characterized by a temperate continental climate, with a mean annual air temperature of 7.8 °C and a mean annual precipitation level of 545 mm. Two different sides were selected, namely, Konstancin-Borowina (52° 03′ 24″ N, 21° 06′ 46″ E) and Konstancin-Zabieniec (52° 03′ 02″ N, 21° 03′ 51″ E). On both sides, the soils were classified as albic podzols (IUSS Working Group WRB 2015) under fresh mixed coniferous species made up predominantly of pine (*Pinus sylvestris* L*.*) trees. Birch (*Betula pendula*), oak (*Quercus robur* L*.*) and black locust (*Robinia pseudoacacia* L*.*) were present at the Konstancin-Borowina, while hazel (*Corylus avellana* L*.*) and rowan (*Sorbus aucuparia* L*.*) were found in the understory layer. Beech (*Fagus sylvatica* L*.*) and spruce (*Picea abies*) were noted at the Konstancin-Zabieniec site, while buckthorn (*Rhamnus cathartica* L*.*) and deadly nightshade (*Atropa belladonna L.*) appeared in the understory layer.

Soil from the two genetic horizons was selected for the research, i.e. the humus horizon (A) and subsurface eluvial horizon (E). Special attention was paid to the characteristic genetic horizon material. The soil samples were collected from the middle of each distinguished soil horizon: the upper 5–10 cm (for both sites) and deeper, at 40–45 cm, in the case of the Konstancin-Borowina (KB) site, and 35–40 cm in the case of the Konstancin-Zabieniec (KZ) site. From the two horizons, disturbed samples were collected to be measured for particle size distribution, total organic carbon (TOC), total nitrogen (N), soil water repellency (SWR) and carbon dioxide efflux. Additionally, three sets of undisturbed soil samples were collected in order to determine soil bulk density and soil moisture retention characteristics.

TOC was measured by a non-dispersive infrared method using a Shimadzu TOC-V analyser with a solid-sample module (SSM-5000A; Shimadzu, Tokyo, Japan) and the nitrogen level was determined by the Kjeldahl method. The particle size distribution was performed using the Bouyoucos method with modifications by Casagrande and Prószyński (the aerometric method) for particles lower than 0.1 mm and the sieve method for particles higher than 0.1 mm (Ryżak et al. 2009). Soil moisture retention characteristics were measured in the laboratory using a standard sand table (pF between 0.4 and 2.0) and the pressure chamber method (pF between 2.7 and 4.2) (Klute 1986). The dry bulk density of each sample was determined by dividing the mass of the particles (oven dried at 105°C) by the volume of an undisturbed soil core sample. The measured basic soil properties are presented in Table 1. According to the USDA classification (Soil Survey Division Staff 1993), the soils were assessed as fine sand, with the exception of the E horizon in the KZ site, which was evaluated as loamy find sand (as it was composed of 15% loam). The total organic carbon content and nitrogen content were higher in the upper horizons. On the other hand, the bulk density values were higher in the deeper horizons. The field moisture content and the permanent wilting point were higher in the upper horizon. After France, Germany and Ukraine, Poland has the largest forest areas in the region, and the soils are typical of those found in pine ecosystems (Hewelke et al. 2015).

**Table 1:** Physical properties of the examined soils from two study sites

Site	Konstancin-Borowina (KB)		Konstancin-Zabieniec (KZ)	
Genetic horizon	A	E	A	E
Depth of sampling (cm)	5–10	40–45	5–10	35–40
Sand (%)	92.0	90.0	94.0	85.0
Loam (%)	8.0	10.0	6.0	15.0
Soil texture	Fine sand	Fine sand	Fine sand	Loamy fine sand
TOC (%)	0.86	0.48	1.91	0.47
N (%)	0.51	0.30	1.02	0.33
Soil bulk density (Mg/m^3^)	1.40	1.48	1.39	1.62
Soil moisture content at pF 2 (%)	15.57	12.97	19.53	11.92
Soil moisture content at pF 4.2 (%)	5.36	4.11	9.25	3.63

### Soil Contamination

Under laboratory conditions, the collected soil material was contaminated with different doses of diesel oil, i.e. D1 and D2 (3000 and 9000 mg of diesel oil per kg of soil, respectively). Before the treatment, the soil samples (500 g of air dry matter in each) were spread in thin layers of about 1 cm on aluminium trays. Then, the soil surface was uniformly dosed with the diesel fuel at a density of 836 g L^−1^ at 15 °C using the sprinkling method. After treatment (5 to 10 min) the soil samples were thoroughly mixed by hand several times. They were then moved to plastic bags, tightly sealed and heated up to 40 °C. Later, the soil samples were mixed again and equilibrated to room temperature for 2 days (Siddiqui and Adams 2002).

### Soil Water Repellency

The soil water repellency was assessed independently for each soil horizon the using Water Drop Penetration Time (WDPT) test. The WDPT test is relatively simple and is the most widespread method (Doerr et al. 2000; Papierowska et al. 2018), having been scientifically evaluated by a widely reproduced classification (Dekker and Jungerius 1990). According to the statement in the literature, the test distinguishes five SWR classes: wettable or non-water repellent, WDPT < 5 s; slightly repellent, WDPT = 5–60 s; strongly repellent, WDPT = 60–600 s; severely repellent, WDPT = 600–3600 s and extremely repellent, WDPT > 3600 s. The SWR was measured for both the uncontaminated soil (0) and the soil treated with diesel oil (D1 and D2). The soil samples were air dried under laboratory conditions at a temperature of 20°C. Then, 20 g of each soil was placed on a Petri dish. Seven drops of distilled water, with a volume of 58 μl in each drop, was applied to the smooth surface of the soil and time it took for the drops to infiltrate was recorded. The median values of the WDPT test were used for further analysis.

### CO_2_ Efflux Measurements

The measurements of soil CO_2_ efflux were taken using a portable infrared gas analyser, the LCpro—from ADC BioScientific Ltd. (Hartley et al. 2008). This chamber method incorporates an enclosed volume (soil hood) to measure the gas exchange associated with the respiration of biomass in soil. The principle of the method is based on measuring the CO_2_ concentration of air entering the hood and comparing this to the concentration of CO_2_ in discharged air passed over the soil surface. Three soil samples, similar to the SWR assessment (control 0, D1 and D2) from each horizon (12 in total) were packed into the soil cores (8 cm high and with a 5.85 cm radius) to the natural bulk density and then were saturated with water for approximately 1 week. The bottom of each sample was protected against evaporation by a rubber cover and was sealed using tape. During the efflux experiment, the surface of the soil samples was exposed to the air and slowly dried through evaporation over 4 weeks. The soil hood was temporarily installed tightly over the soil surface during the evaporation process (to ensure proper measurement conditions inside the hood, the rubber o-ring between hood and core was applied) and CO_2_ was recorded for approximately 20 min when a steady state air flow had been achieved over the soil surface. The soil samples were weighed at the same time as the CO_2_ measurements were taken to determine their average water content. At the first stage of evaporation, the CO_2_ efflux was measured once every 12 h. Then, after approximately 1 week, the measurements were taken approximately once per day or once per 2 days. The efflux measurements were taken until the changes in soil moisture were negligible. Under laboratory conditions, the soil samples were air dried for 1 week and finally, the mass oven dried at 105°C was recorded to determine changes in moisture content during the efflux experiment.

### Statistical Analysis

The analysis of variance was applied to evaluate the influence of soil depth and the level of diesel oil contamination on CO_2_ efflux. The statistical significance was accelerated by the analysis of variance (the two-way ANOVA and the LSD mean test). Differences between the mean values of the soil groups were evaluated using Tukey’s test (*P* < 0.05). In order to establish the relationship between CO_2_ efflux and soil moisture content, the following Gaussian non-linear equation was used:

1 $$ {F}_{\mathrm{CO}2}\left(\theta \right)=a\kern0.5em \mathit{\exp}\;\left[-0.5\kern0.5em {\left(\frac{\theta -b}{c}\right)}^2\right] $$

*F*_CO2_: soil efflux of CO_2_ [μmol m^−2^ s^−1^],
*θ*: soil moisture content [m^3^ m^−3^],
*a*, *b*, *c*: empirical parameters of the equation.

All statistical analyses were conducted using Statgraphics Centurion version XVI (StatPoint Technologies, Inc., 2009).

## Results and Discussion

### Potential Soil Water Repellency

**Fig. 1:**
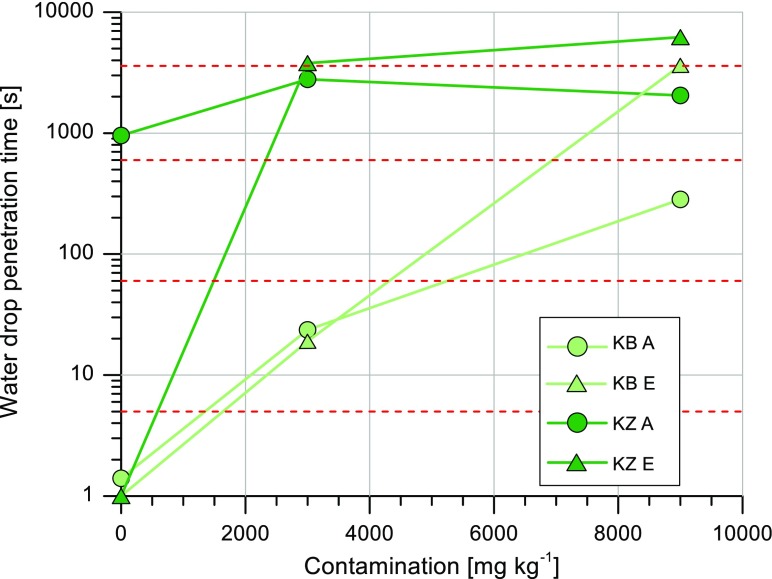
Potential soil water repellency (SWR) classes at different levels of diesel oil contamination for KB and KZ sites in A and E soil horizons. SWR classes (dashed lines): wettable, WDPT < 5 s; slightly repellent, WDPT = 5–60 s; strongly repellent, WDPT = 60–600 s; severely repellent, WDPT = 600–3600 s and extremely repellent, WDPT > 3600 s

Two types of unpolluted soils with different initial non-wetting characteristics were selected from the upper horizon: hydrophilic soil at the KB site and hydrophobic soil at the KZ site. The potential soil water repellency of the studied soils, expressed as WDPT classes, is shown in Fig. 1. In the four analysed horizons, contamination increased hydrophobicity. There are considerable differences in the laboratory results between each of the WDPT classes at the two study sites. In both (A and E) horizons of the KB site, the uncontaminated samples were found to be inherently wettable. The D1 samples were all slightly repellent in both horizons. The D2 sample was strongly repellent in the upper horizon, while the same contaminated soil sample was severely repellent in the deeper horizon. At the KZ site, the uncontaminated soil samples were found to be inherently severely repellent in the upper horizon, and for deeper horizon, they were naturally wettable. The subsamples D1 and D2 were found to be mostly severely repellent and extremely repellent in both of the KZ horizons. All samples were water repellent following contamination by hydrocarbons, evidently confirming the hypothesis that hydrocarbon contamination induces water repellency in inherently wettable sandy forest soils, while naturally severely repellent soil remained in the same class of severe repellency. This result was also observed in the soils underneath vegetation in pine forests studied by Zavala et al. (2009), which were always found to be water-repellent with wettability only being observed in some bare areas. This suggested that pine trees have high potential for inducing SWR. The significant correlation between soil organic matter and WDPT class has been reported for forest soils underlying cork oaks, heaths, eucalyptus and various pine species (Mataix-Solera and Doerr 2004; Rodríguez-Alleres et al. 2007). The authors argued that the difference in SWR is due to the different chemical compositions of the plant tissues, particularly the contribution made by resins, waxes and aromatic oils. Hydrocarbon contamination induces water repellency in inherently wettable tropical sandy soils and reduces soil moisture retention at low suction (˂ 100 kPa) in laboratory-contaminated soils, but the effects were inconsistent for field samples (Takawira et al. 2014) due to the presence of hydrophobic long-chain aliphatic and aromatic compounds in petroleum hydrocarbons.

Klamerus-Iwan et al.’s (2015) and Błońska et al.’s (2016) researchs found that the amount of oil introduced into the soil is correlated with the enzyme activity, biomass and density of earthworms, as well as with the soil water content and capillary water capacity. The initial degree of soil water repellency in a laboratory investigation on moisture migration through oil-contaminated hydrophobic soils was also an important factor in the study of Quyum et al. (2002).

### CO_2_ Efflux

**Fig. 2:**
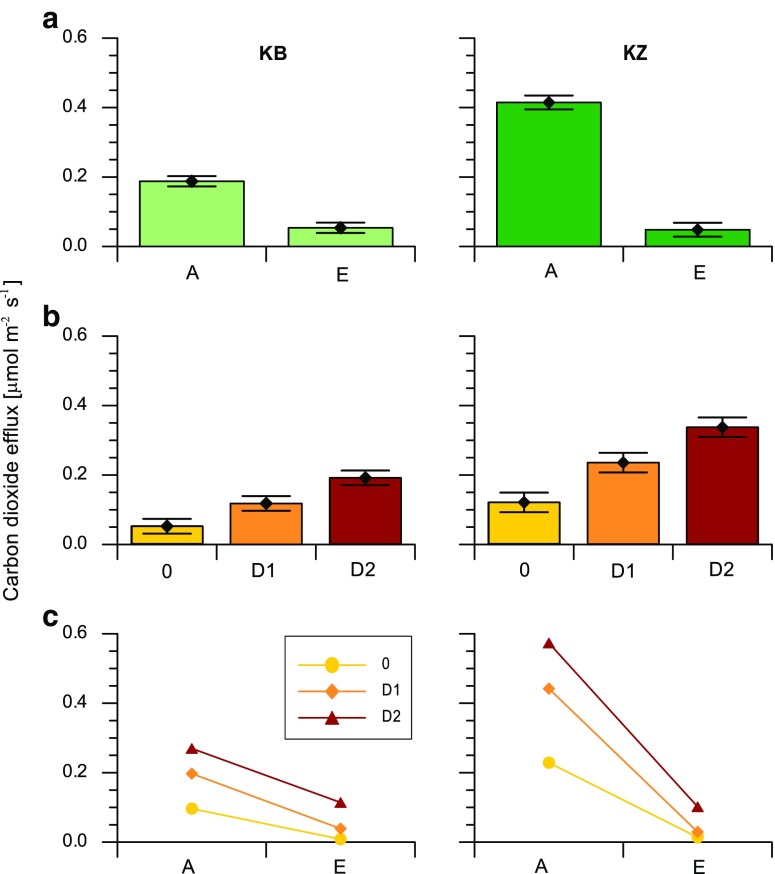
Results of the ANOVA analysis of carbon dioxide efflux for the different study sites: KB—left, KZ—right, for two soil horizons (A and E) and three levels of diesel oil contamination (0, D1, D2)

A multiple-comparison analysis of variance test was carried out for the two different horizons of both study sites and for the different contamination levels of D1, D2 and control 0, which is presented in Fig. 2. It was found that both factors, i.e., soil depth and contamination level, had a statistically significant influence on CO_2_ efflux at the 95% confidence level. The main effect of depth was significant for both profiles, and the CO_2_ efflux rate in the upper horizons was considerably higher than the efflux rate in the deeper horizons: it was more than three times higher for KB, and more than 10 times higher for KZ. The biggest mean value was observed in the upper horizon of soil at the KZ site (0.4146 μmol m^−2^ s^−1^), which was categorized into the severely repellent class for all levels of diesel oil contamination (Fig. 2a). The mean value and 95% confidence intervals of the CO_2_ efflux at each of the three contamination levels are presented in Fig. 2b. The mean CO_2_ efflux value was quite a lot lower in uncontaminated soils and increased with the level of contamination. The CO_2_ efflux of the uncontaminated soils and the D1- and D2-contaminated soils, increased nearly twofold with each level at the KB and KZ sites, and three homogenous groups were identified for each contamination level at the two study sites. Figure 2c presents the mean values for the individual measurements of the different levels of diesel oil pollution in each horizon.

According to research conducted by Kutsch et al. (2010), in an undisturbed old-growth deciduous forest in Germany, soil depth played a vital role with regard to the CO_2_ emission rate. Consequently, the TOC content was found to be higher in the upper horizons and lower in the deeper horizons, thus making a big difference in CO_2_ efflux between the upper and lower soil horizons. The results of this research confirm the findings of Dickinson et al. (2005), who showed that the depth of the soil profile has a significant effect on CO_2_ emissions; they are higher in the top 5–10 cm horizon of the soil, but get lower as the horizons get deeper. This is attributed to microbial activity. CO_2_ emissions in petroleum-contaminated sandy soils were higher than in uncontaminated sandy soils, which can probably be attributed to the inhibition of microbial biomass by hydrocarbon products (Labud et al. 2007). Moreover, Kaur et al. (2015) also carried out experiments related to the effect of oil contamination on soil respiration, finding a significant difference in CO_2_ emissions between contaminated and uncontaminated soils, which is due to microbial activity.

**Fig. 3:**
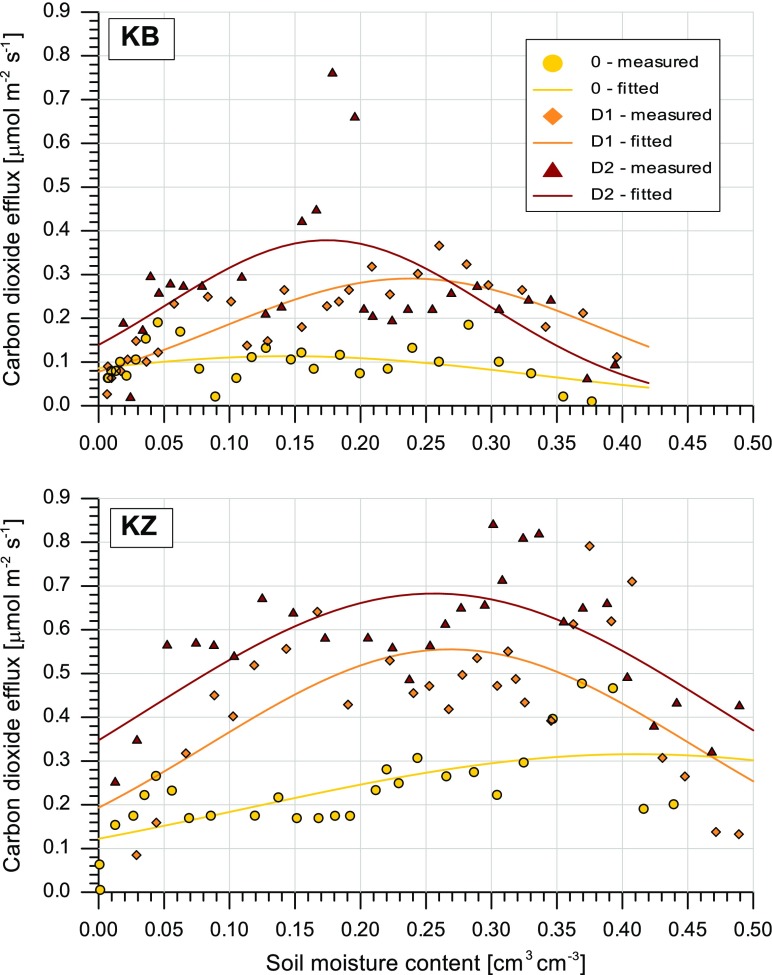
Relationship between CO_2_ efflux and the moisture content of the analysed upper horizon (5–10 cm) soils

Two upper horizon (A) samples from both study sites were chosen to analyse the influence of soil moisture content on CO_2_ efflux. In case of E horizons the impact of moisture content on CO_2_ efflux was insignificant. The parameters of Eq. (1) and the coefficient of determination are presented in Table 2. Figure 3 presents the measured and calculated upper soil horizon data for the relationship between CO_2_ efflux and changes in soil moisture content for both the KB and KZ sites. In the uncontaminated soils, CO_2_ efflux is fairly constant and less scattered across a whole range of changes in moisture content in comparison to soil contaminated with diesel oil. However, in the untreated repellent soil (KZ site), the maximum efflux (parameter *a* = 0.31 μmol m^−2^ s^−1^) was obtained at a moisture content of approximately 41%, while in the naturally non-repellent soil (KB site) the maximum CO_2_ emission (*a* = 0.11 μmol m^−2^ s^−1^) was estimated when the soil was relatively dry (14.4%). Despite the similar physical properties of both soils, it can be observed that severely repellent soil induces as much as double the CO_2_ efflux to that of soil classified as wettable. Such a large difference in the greenhouse gas emission rate at constant room temperature is probably correlated with the TOC and N content of the uncontaminated soils (Table 1). In both soils, the contamination treatment causes a significant increase in CO_2_ emissions within the middle range of changes in moisture content. This is expressed in the value of parameter c, which represents the majority of the dispersion of the Gaussian type curve. In the essentially non-repellent soil, the moisture content range that was favourable for CO_2_ efflux decreased as the amount of diesel oil in the soil increased. Simultaneously, the maximum rate of CO_2_ efflux increased from 0.29 μmol m^−2^ s^−1^ for contamination level D1 to approximately 0.378 μmol m^−2^ s^−1^ for contamination level D2. In the soil from the KZ site, increasing the amount of contaminant did not have a significant influence on the value of the c parameter. However, the range of changes in moisture content conducive for gas emission in KZ soil was relatively wider than in KB soil (higher c parameter values). Based on the data presented in Fig. 3, it can be concluded that CO_2_ efflux from the investigated soils took place at a similar rate whether the moisture content was low or whether the soil was saturated with water, and was not dependent on the soil contamination treatments. The obtained data generally agree with the findings of Luo and Zhou (2006), i.e. that soil water content indirectly affects CO_2_ efflux by limiting oxygen diffusion at higher water contents and the diffusion of soluble substrates at low water contents. The results from the field observations of Chayawat et al. (2012) indicated that CO_2_ efflux from soil appeared to be influenced by soil water content following rainfall events.

**Table 2:** Parameters of Eq. (1), describing the relationship between CO_2_ efflux and soil moisture content and coefficients of determination

Site	Treatment	Equation (1) parameters			Coefficient of determination *r*^2^
		*a*	*b*	*c*	
KB	0	0.1136	0.1445	0.1942	0.149
	D1	0.2910	0.2381	0.1468	0.726
	D2	0.3786	0.1743	0.1233	0.328
KZ	0	0.3156	0.4104	0.2980	0.441
	D1	0.5553	0.2686	0.1848	0.424
	D2	0.6829	0.2561	0.2203	0.522

## Conclusions

The current study investigated the CO_2_ efflux and water repellency of fine sand and loamy fine sand contaminated in a laboratory, derived from parent material in forest soils in Poland. The soils were inherently wettable and inherently severely repellent and consisted predominantly of sand fraction size (85–94%). Hydrocarbon contamination has the capacity to induce water repellency. The initially water-repellent soils were found to have a bigger CO_2_ efflux. Two different factors, i.e. soil depth and level of contamination, have a significant effect on the CO_2_ efflux rate in soil. The CO_2_ efflux rate turned out to be considerably higher in the upper horizons of soil than the deeper horizons, which could be attributed to the type of vegetation, the root zone and microbial community activities, where there is more organic matter. The difference between hydrocarbon-contaminated soils and uncontaminated upper horizon soils, in terms of CO_2_ efflux, was nearly double. A relationship was found between soil moisture content and CO_2_ efflux in upper intact and polluted soil horizons. The contamination treatment within the mid-range of changes in moisture content caused a significant increase in CO_2_ efflux. The maximum value of CO_2_ efflux was near to the pF value of the field capacity measured for uncontaminated soils. The main conclusion of our study is that, soil water repellency and its interaction with soil moisture under the effects of hydrocarbon contamination on soil should be included in the assessment of hydrocarbon-contaminated soils. The role of wetting in polluted soil can facilitate treatment optimization; however, additional studies are needed to improve our understanding of soil respiration processes in field studies.
